# The Ice Treatment

**Published:** 1889-11-16

**Authors:** 


### THERAPEUTICS.

### THE ICE TREATMENT.

A short time ago we mentioned in the "Retrospect" cases treated by ice to the spine by Dr. Kinnear, of Philadelphia. These ailments were insomnia, herpes zoster, and spasmodic croup. The ice treatment, however, it appears, is not to be confined to the class of maladies mentioned by Dr. Kinnear. Dr. D. B. Lees, M.D.Cantab, physician and lecturer on medicine, St. Mary's, has contributed a paper to the *Lancet* on the treatment of pneumonia by the ice-bag. A case is detailed in which immediate and final arrest of the pyrexial process followed the application of the ice-bag. And another case is related in which immediate arrest of the pneumonic process followed the application of the ice-bag and a relapse followed its removal. A third case is recorded similar to the last, a second fall occuring when the ice-bag was reapplied. The use of cold applications to the chest was advocated by Niemyer twenty years ago. But he used cold compresses, which Dr. Lees thinks very inferior to the ice-bag. The ice-bag retains its cold until all the ice is melted—hence it produces a more permanent local impression. Dr. Lees states he has never found any objection to the use of the ice-bag, which is usually pleasant to the patient. The bag is applied over the part of the lung affected. In one case the affected site was tightly strapped and the bag applied over the strapping; but in other cases no mention is made of strapping. A discussion on this method of treating pneumonia took place at the Harveian Society a few weeks ago, when Dr. Goodhart said that for some months past he had used no other application than the ice-bag in cases of acute pneumonia. But the views of other speakers were not altogether in favour of the treatment. While Dr. Goodhart obtained a good result in eight cases, in seven the result was doubtful, and in three cases symptoms of collapse were produced, which, however, subsided on the removal of the ice-bag. Dr. Sturges remarked that in a case of lobular pneumonia in the Westminster Hospital, under Dr. Donkin, the ice-bag was successful, but he doubted if the evidence were yet sufficient for us to say that it arrested pneumonia. It is difficult to eliminate the question of the natural crisis of pneumonia having coincided with the application of ice. Dr. Sidney Phillips observed an objection to the ice-bag was that on its removal the temperature again rose sometimes to a higher degree than before. Dr. Tirard remarked that although comfort was increased the disease was not shortened. The treatment of pneumonia by cold baths, as recommended some time back by Juergensen, did not find much favour, and we doubt if the treatment by the ice-bag will hold its own. For the cold bath not unfrequently produced alarming depression, and so may the ice-bag. Pneumonia is divisible into several varieties—viz., acute pneumonia, secondary, lobular, chronic or interstitial. In acute pneumonia, for which the ice treatment is more especially advocated, the symptoms may continue with often increasing severity up to the end of the first week, or, perhaps, a little longer, when an improvement usually occurs. This improvement may take place quite suddenly, and the malady terminates by *crisis*. In other instances the improvement is more gradual, the termination being by *lysis*. Complete recovery is common in young and healthy people, and the termination is often by crisis. This may occur as early as the third day, or it may occur on the fifth, sixth, or seventh day. When it so occurs neither ice nor any other treatment can be credited as the reason why. The ice-bag treatment of pneumonia must therefore, we think, be regarded as *sub judice*, even when viewed in the most favourable aspect.

